# Nicolau syndrome following Intramuscular Diclofenac Injection: a case report and review of the literature

**DOI:** 10.1093/jscr/rjad224

**Published:** 2023-04-28

**Authors:** Yaser M Ata, Mohamed B Ahmed, Fatima S Al-Mohannadi, Fatima A Al-Jassim, Azhar Shabbir

**Affiliations:** Urology Department, Hamad General Hospital, Hamad Medical Corporation, Doha, Qatar; Plastic Surgery Department, Hamad General Hospital, Hamad Medical Corporation, Doha, Qatar; College of Medicine, QU Health, Qatar University, Doha, Qatar; Plastic Surgery Department, Hamad General Hospital, Hamad Medical Corporation, Doha, Qatar; Urology Department, Hamad General Hospital, Hamad Medical Corporation, Doha, Qatar; Acute Care Surgery Department, Hamad General Hospital, Hamad Medical Corporation, Doha, Qatar

**Keywords:** Diclofenac Sodium, Nicolau syndrome, intermuscular injection, Embolia cutis medicamentosa, livedo-like dermatitis

## Abstract

Nicolau syndrome (NS), also referred as embolia cutis medicamentosa and livedo-like dermatitis, is an uncommon complication followed by drugs administered intramuscularly, intraarticularly or subcutaneously. In this case report we present a case of a 65-year-old lady who had a single dose of diclofenac sodium as an intramuscular injection in her left buttock due to back pain that led to developing what known as NS. She was treated with surgical debridement, drain insertion and skin approximation with antibiotics for 2 weeks with daily sterile dressing. The wound healed completely with scarring. NS is a preventable outcome, thus, proper procedures and precautions should be taken during intramuscular medication administration. Healthcare providers should avoid unnecessary injections, be familiar with the complication and consider it as a potential diagnosis for severe localized pain after any injection.

## INTRODUCTION

Nicolau syndrome (NS), also referred as embolia cutis medicamentosa and livedo-like dermatitis, is an uncommon complication followed by drugs administered intramuscularly, intraarticularly or subcutaneously. It was first noted in 1920s after a patient received bismuth salts injections for syphilis [1]. However, it is also observed after injecting numerous medications, such as non-steroidal anti-inflammatory drugs, Penicillin, local anesthetics, corticosteroids and vitamins [2]. It has been hypothesized that this adverse reaction occurs when medications cause vasospasms and perivascular inflammation leading to the development of skin discoloration and pain at the injection site. This is progressed to an erythematous patch, livedoid reticular pattern, hemorrhagic lesions and ultimately skin or deeper tissue necrosis [3].

## CASE PRESENTATION

A 65-year-old lady who is known to have Diabetes mellites, Hypertension and Hyperthyroidism on medication presented with 4-week history of blackish skin discoloration at the lateral upper quadrant of her left buttock. Before this presentation the patient had an episode of a lower back pain in which she received Diclofenac injection 75 mg by a family member. She had immediate and constant pain at the injection site but considered it normal. The following few days the patient started complaining of painful swelling and blanching redness at the injection site, the patient did not interfere however the swelling became progressively more painful, prominent and gradually increasing in size with surrounding induration, her daughter noticed a blackish discoloration ⁓5 × 5 cm surrounded by redness and induration.

**Figure 1:**
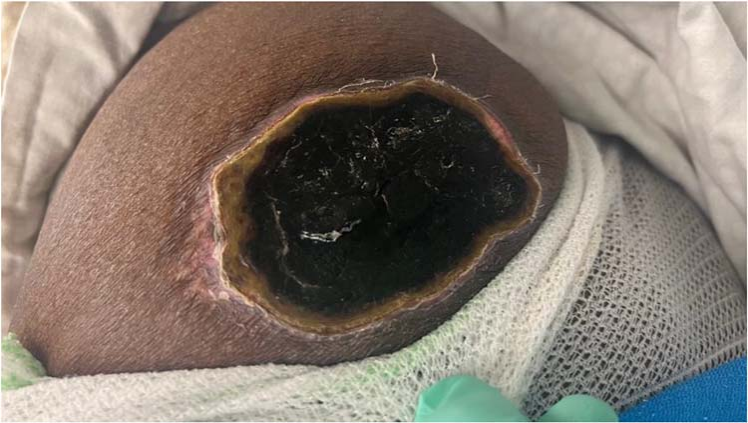
Left gluteal region showing necrotic patch 4 weeks following Intramuscular Diclofenac Injection.

In the emergency department the patient had left gluteal aching pain no aggravating factors relived mildly with oral analgesics, there was no other symptoms. By examination, there is a non-tender indurated blackish patch ⁓8 × 4 cm at the center of the left buttock surrounded by reddish area (Fig. 1). The patient was vitally stable, laboratory tests were within normal range. Ultrasound soft tissue showing soft tissue thickening and inhomogeneous areas of hyper echogenicity of the left gluteus, measures ⁓3.5 cm in depth; concerning for hematoma, no liquified collection.

**Figure 2:**
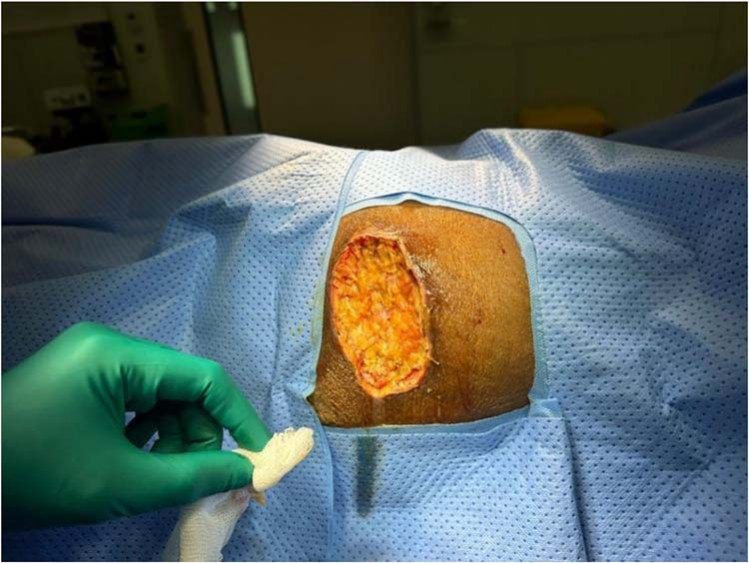
Intraoperative image of left gluteal region post-debridement of the soft tissue necrosis until reaching healthy tissue.

The patient was sent to the theater for excision of the necrotic patch under laryngeal mask airway anesthesia, Lateral position left side up, excision of the necrotic patch done, with finding of extensive Fibrotic tissue with multiple pockets of seropurulent fluid. All the fibrotic tissue was excised until healthy margins of subcutaneous fat achieved (Fig. 2).

**Figure 3:**
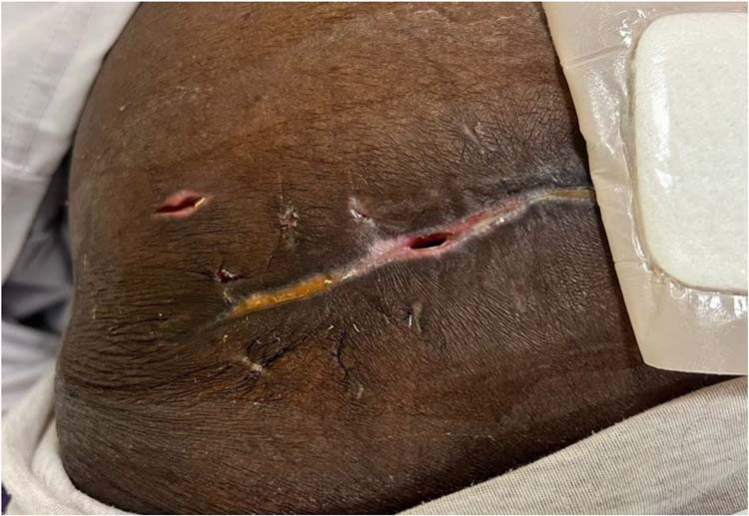
Post-operative image of the left gluteal region 3 weeks after debridement and closure of the wound.

Approximation of the subcutaneous tissue was done and finally skin closure with insertion of mini vac drain. After 1 week inpatient the drain was removed (total drainage = 70 ml), and the patient discharged home. Came after 2 weeks for wound assessment and removal of the stitches (Fig. 3).

## DISCUSSION

NS is a widely recognized, although uncommon, adverse effect that can occur mainly as a result of intramuscularly, intraarticularly or subcutaneous injections. Usually, after an injection, people experience a whitening of the skin and pain in the area around the injection site followed by erythema. After that, the affected area then becomes hemorrhagic and eventually necrotizes, leaving a scar as it heals [4]. Several reports have described similar reactions after the injection of various other substances, suggesting that this phenomenon may not be directly related to the specific drug that was administered [5]. Although there have been various theories to describe the pathogenesis published in the literature, the exact cause of the disease is unknown [6] (Table 1).

**Table 1:** Summary of the demographics and clinical findings of patients with NS post Intramuscular Diclofenac Injection in the literature

Study	Gender	Age (years)	Location of intramuscular injection	Admission post injection	Considerable clinical findings	Management	Follow-up
Kılıç* et al.* [10]	Female	45	*Right gluteal region*	3 weeks	Skin necrosis ⁓15–20 cm	Surgical debridement and Antibiotics due to secondary bacterial infection	Complete healing in 12 weeks
Bhanja *et al.* [2]	Male	26	*Left gluteal region*	4 days	Tender place with necrosis ⁓7 × 5 cm	Antibiotics given to prevent secondary bacterial infection and surgical debridement of the necrotic tissue	
Lie *et al.* [5]	Male	58	*Right gluteal region*	2 weeks	Necrotic skin patch 10 × 7 cm	Multiple surgical debridements done and a partial-thickness skin graft was performed	Complete healing with large scar
Panariello *et al.* [11]	Female	65	*Left gluteal region*	4 weeks	skin patch 4.2 × 2.2 cm	*Treated with zinc oxide paste, applied to the affected area twice a day*	
Nayci *et al.* [12]	Female	77	*Right gluteal region*	Not mentioned	5 × 4-cm-size, well-defined ecchymotic plaque surrounded by a livedoid, reticular erythematous patch with ruptured blister particles	Conservative with local wound care.	Within the following weeks a necrotic ulcer developed and atrophic scar remined
Kim *et al.* [3]	Female	69	*right gluteal region*	inpatient	*The skin showed erythematous maculae and a livedoid violaceous patch with dendritic extensions by the 10th day post injection the skin turned dark and changed into eschar*	Multiple surgical debridements and skin covering with full-thickness skin graft	Skin healed with residual scar
Sasmal *et al.* [1]	Male	60	*Left gluteal region*	Few days	Extensive areas of necrosis covered with black eschar present over the bilateral gluteal region and lateral upper thighs (left more than right) extending	Serial extensive wound debridement sparing the underlaying muscles with split-thickness skin graft	Healthy skin graft with scar

The syndrome occurs more frequently in children, particularly those under 3 years old. This is because the risk of artery embolism may be higher in this age group due to the smaller size of the vascular segments involved [7]. NS is a preventable complication. While it is not possible to predict when NS may occur, the risk of tissue damage can be minimized by administering the injection to the superolateral region of the gluteal muscle using a needle that is long enough to reach the muscle [8]. There is no specific management for NS lesions. The affected area should be cleaned and dressed and painrelief measures should be taken. Preventing infection is important and topical steroids and thrombolytic agents may be applied depending on the severity of the lesions. In cases where there is no skin or soft tissue necrosis, anticoagulant therapy and frequent monitoring may be helpful. However, in cases of severe tissue death and ulceration, as seen in our case, extensive debridement of dead tissue and wound closure options (direct closure, secondary healing, graft, flap) should be assessed [9]. In our case, the wound was more severe as the patient initially neglected the lesion thinking it will resolve spontaneously until the lesion deteriorated reaching an extensive soft tissue necrosis and superimposed infection with deep pockets of seropurulent fluid, which led to an extensive debridement and inserting a drain for continuous drainage of the pus with adjuvant systemic antibiotics for 14 days. Our management was different from the literature as the aim of inserting a drain was to minimize seroma collection in the debrided area and enhances faster healing of the tissue.

## CONCLUSION

The main focus of management is to prevent the occurrence of NS. This can be achieved by taking simple precautions and following recommended techniques for administering intramuscular drugs, which can effectively eliminate the risk of this serious adverse event.

It is important for healthcare providers to be familiar with this complication and to avoid administering intramuscular injections when they are not necessary. Additionally, physicians should keep NS in mind as a potential diagnosis for anyone who experiences severe localized pain after receiving an injection of any substance into a muscle.
